# Recent Advancement in Anticancer Activity of *Clinacanthus nutans* (Burm. f.) Lindau

**DOI:** 10.1155/2021/5560502

**Published:** 2021-05-26

**Authors:** Chung-Ming Lin, Hsin-Han Chen, Chi-Wen Lung, Hui-Jye Chen

**Affiliations:** ^1^Department of Biotechnology, Ming Chuan University, Taoyuan 33348, Taiwan; ^2^Division of Plastic and Reconstructive Surgery, Department of Surgery, China Medical University Hospital, Taichung 40402, Taiwan; ^3^Department of Creative Product Design, Asia University, Taichung 41354, Taiwan; ^4^Department of Nursing, Asia University, Taichung 41354, Taiwan; ^5^Graduate Institute of Biomedical Sciences, China Medical University, Taichung 40402, Taiwan

## Abstract

*Clinacanthus nutans* is a traditional medicinal herb that is applied for the therapy of snake bites, skin infection, herpes infection, burns, scalds, dysentery, and diabetes. *Clinacanthus nutans* is also used to treat several cancers, including breast, cervical, colon, gastric, head and neck, liver, lung, pancreatic, and skin cancers, as well as lymphoma and leukemia; however, the underlying mechanisms of its anticancer activity remained undetermined. We searched PubMed and Google with key words “*Clinacanthus nutans* and cancer” and collected recent papers of *Clinacanthus nutans* with anticancer activity. We focused on the preparation, effects, and action mechanisms of *Clinacanthus nutans* extracts on various types of cancers. We hope that this mini review can help update our knowledge about active components, effects, and molecular mechanisms of extracts from this promising herb *Clinacanthus nutans* for ongoing studies and speed up its clinical application in the future.

## 1. Background

*Clinacanthus nutans* (Burm. f.) Lindau (*C. nutans* for abbreviation thereafter) is a perennial herb that belongs to the Acanthaceae family, which consists of many medicinal species of plants with high value [1]. For detailed description of morphology and characteristics of this plant, please refer to previous reviews [1–3]. *C. nutans* is a vegetable and traditional herb in Southern Asian countries such as Thailand, Indonesia, and Malaysia [4]. This plant is usually used for the treatment of snake bites, skin infection, burns, scalds, hurts, dysentery, diabetes, virus infection, and even cancers [5–7].

A flurry of research indicated that *C. nutans* possesses various biological activities including antimicrobial, antiviral, antioxidant, antidiabetic, anti-inflammatory, immunomodulatory, antihyperlipidemic, and anticancer activities [2]. *C. nutans* now becomes a multifunctional plant that draws attention of scientists in various research areas. A bunch of reviews on the detailed phytochemistry and pharmacology of *C. nutans* have been provided [1, 4, 8, 9]. Here we intend to focus on recent progress of the anticancer activity of *C. nutans*. We searched PubMed for most related literature by the key words “*Clinacanthus nutans* and cancer” from the year 2015 to the year 2020, and 27 articles were selected, including 5 reviews. Those articles that are not related to anticancer activity were sacrificed due to the scope of this mini review. Some dated studies were also included in order to have a more complete story of *C. nutans* on various cancers. We also performed Google search and a thesis and a conference paper were also retrieved, giving that they have contribution on the studies of anticancer activity of *C. nutans.*

Cancer patients that were subjected to oral administration of *C. nutans* extract, blended with fruit juice to reduce the bitterness, have been shown to be recovered from a variety of cancers [10, 11]. Toxicity studies have shown that *C. nutans* extracts were more or less nontoxic [9]. These studies highlight the potential of *C. nutans* for cancer therapy. To keep track of the progress of anticancer activity of *C. nutans* and point out a new direction for further research and future clinical application, we aimed to review up-to-date publications about the effects of *C. nutans* on different types of cancer, including breast, cervical, colorectal, gastric, liver, lung, head and neck, skin, and pancreatic cancers, as well as lymphoma and leukemia, primarily based on cell and animal studies. A novel way of extract package such as nanoencapsulation has also been tried in a cell-based study [12]. Preparation of extract and the investigation of their anticancer activities and possible underlying mechanisms of *C. nutans* extracts are listed in Table 1. Two interesting studies about combination therapy of *C. nutans* extracts with current anticancer drugs are listed in Table 2.

## 2. Effects and Action Mechanism Studies of *C. nutans* Extracts on Cancers

### 2.1. Breast Cancer

According to the literature, the different parts of whole plants of *C. nutans*, including leaves, stem, roots, and even bark, have been used for combating diseases [13]. Therefore, Teoh and colleague isolated methanol and ethyl acetate extracts from *C. nutans* roots and tested antiproliferative as well as apoptosis-inducing activities of these extracts on MCF-7 human breast cancer cells. Both extracts inhibited proliferation of MCF-7 cells with the IC_50_ of 35 and 30 *μ*g/mL for methanol and ethyl acetate extracts, respectively. Induction of apoptosis was evidenced by chromatin condensation and downregulation of BCL2. However, the expression level of BAX was not changed. Further evidences showed that ethyl acetate extract was able to decrease the mitochondrial membrane potential by JC-1 staining. GC-MS identification revealed that these root extracts are rich in terpenoids and phytosterols, so their anticancer activities need to be further analyzed [14].

In addition to root extract, methanol extract from leaves of *C. nutans* has been isolated and the antioxidant and antitumor activities in 4T1 tumor-bearing mice of breast cancer using low-dose concentration (200 mg/kg) and high-dose concentration (1000 mg/kg) of extracts were determined. Both dosages of extract significantly decreased the blood nitric oxide (NO) and malondialdehyde (MDA) levels. High-dose extract effectively reduced the number of mitotic cells, tumor weight, and tumor volume of 4T1 breast cancer model mice. However, extract dosage as high as 1000 mg/kg did not elicit adverse effect and inflammatory response. These data indicate that methanol extract from leaves of *C. nutans* would be potential for the therapy of breast cancer due to its antitumor activities and nontoxicity at given dosages [15].

Besides, crude methanol extract of bark powder from *C. nutans* was isolated and used for further fractionation by chromatography, and six fractions were obtained. These fractions were then tested for their cytotoxicity toward MDA-MB-231 and MCF-7 breast cancer cell lines by dimethylthiazol diphenyltetrazolium bromide (MTT) assay. Two fractions, A12 and A17, which harbor sulfur-containing compounds, including entadamide C and clinamide D, were found to have strong cytotoxic activities. The molecular target of these two compounds was accessed to be caspase-3 by molecular docking simulation, which will pave the way for the design and development of more effective drugs based on structures of entadamide C and clinamide D against breast cancers and other cancers [16].

Recently, Ismail and his colleagues obtained 80% methanol extract from *C. nutans* leaves and subsequently the crude extract was fractionated by n-hexane, dichloromethane, chloroform, n-butanol, and water. Then a series of analyses including total phenolic content (TPC), total flavonoid content (TFC), and antioxidant and antiproliferation activities were performed on MCF-7 breast cancer cells and normal MCF 10A cells. Crude extract (CN-Crd) itself had the strongest antioxidant scavenging activity, while water fraction (CN-Aqu) possessed the highest amount of TPC and TFC. Hexane fraction (CN-Hex) displayed the highest antiproliferative effects on MCF-7 cells, with the IC_50_ of 50.34 ± 0.11 *μ*g/mL, followed by the dichloromethane fraction (CN-Dcm) (IC_50_: 65.95 ± 0.14 *μ*g/mL). As dichloromethane fraction (CN-Dcm) had the highest selectivity index (SI = 1.48), it was chosen for further analyses. GC-MS identification indicated that linolenyl alcohol (29.10% at 12.023 min) and palmitic acid (23.84% at 11.133 min) are the most abundant among 14 compounds. Molecular docking studies showed that p53-binding protein Mdm-2 is the cellular target of these two major components. Their data suggested that methanolic extract from *C. nutans* leaves possesses potential antiproliferative activities, as reported by other laboratories [17, 18]. Besides, Quah et al. reported the antiproliferative activity of methanolic extract from *C. nutans* leaves on MDA-MB-231 cells with IC_50_ of 18.67 *μ*g/mL. However, the same extract was much less toxic to normal 3T3 cells. Further analyses showed that the antiproliferative effects were possibly attributed to the inhibition in activities of CYP3A4 and CYP2E1, two liver enzymes for detoxifying drugs [19].

### 2.2. Cervical Cancer

To combat cervical cancer, petroleum ether, ethyl acetate, and methanol crude extracts of *C. nutans* leaves and stems were produced and subsequently isolated by bioassay-guided fractionation. These fractions were then tested for antioxidant and antitumor activities against HeLa cervical cancer cells. Among these extracts, petroleum ether extract displayed the strongest cytotoxic activity against HeLa cells with IC_50_ of 18.0 *μ*g/mL. Petroleum ether extract also possessed the highest radical scavenging activity. These data suggest that active compounds in *C. nutans* extracts, especially petroleum ether extract, are health-promoting reagents with antioxidant activity and potential cytotoxic activity to cancer cells [20]. Another laboratory reported the further fractionation of *C. nutans* methanol extracts by hexane (methanol-hexane), dichloromethane (methanol-dichloromethane; DCM), and water (methanol-water) and evaluated the effects of these extracts on various biological activities, including cell proliferation, apoptosis, and cell cycle progression to HeLa cells. They found out that all extracts displayed antiproliferative activities to HeLa cells, and DCM extract had the highest activity with the IC_50_ to be 70 *μ*g/mL at 48 hours. DCM extract also induced apoptosis and cell cycle arrest at S phase. GC-MS analysis revealed that at least 28 compounds exist in DCM extract and most of them are fatty acids. The anticancer effects of these compounds or combination of compounds are pending [17].

Yusmazura et al. isolated aqueous and methanol extracts from *C. nutans* leaves and tested their cytotoxicity to HeLa cells. They found out that aqueous extract showed great cytotoxic effects (IC_50_ = 13 ± 0.82 *μ*g/mL) and induced apoptosis of HeLa cells, while both extracts were not toxic to normal kidney Vero cells [21]. Moreover, Ghasemzadeh and his colleagues compared the methanol extracts of leaves and buds of different plant ages, including one-month, six-months, and one-year-old *C. nutans*. The six-month-old buds contain highest total flavonoid (TF) (6.32 mg/g dry weight) and total phenolic (TP) compounds (18.21 mg/g dry weight). Highest contents of caffeic acid (0.307 mg/g dry weight) and gallic acid (5.96 mg/g dry weight) phenolic acids were detected in extracts of one-year and six-month-old buds, respectively. The activity of chalcone synthase (CHS, EC 2.3.1.74), a key enzyme for flavonoid production, was the highest in six-month-old buds (9.5 nkat/mg protein). The 1, 1-diphenyl-2-picrylhydrazyl (DPPH) activity was the highest in the extract of one-year-old buds with 50% of free radical scavenging (IC50) values of 64.6 *μ*g/mL. However, the ferric reducing antioxidant power (FRAP) activity was higher in six-month-old buds (488 *μ*M of Fe(II)/g). MTT assay showed that the extract of six-month-old bud significantly decreased cell viability of HeLa cells with IC50 of 56.8 *μ*g/mL. These analyses indicate that six-month-old buds of *C. nutans* harbor substantial amount of secondary metabolites, providing valuable antioxidant and anticancer compounds [22].

### 2.3. Colorectal Cancer

Leaf extracts from five different reagents, including ethanolic, methanolic, 50% ethanolic, 50% methanolic, and water extracts, of *C. nutans* were produced and tested on HCT-116 colorectal carcinoma cells, and CCD-18Co normal colon fibroblasts. Extract concentrations at 200 and 100 *μ*g/mL showed no significant cytotoxicity on tested cell lines. Further fractionation of methanolic extract (CN-M) by silica gel flash column chromatography showed that fractions 3, 4, 14, and 16 displayed significant cytotoxicity to HCT-116 cells at the concentration of 200 *μ*g/mL. Among them, fraction 14 showed highest growth inhibition activity (84 ± 1.1% at 100 *μ*g/mL) [23, 24]. Besides, ethanol extract and thereafter the hexane, ethyl acetate, and aqueous fractions of *C. nutans* leaves were obtained by another laboratory and their biological activities were tested on HCT-116 cells. *C. nutans* ethyl acetate fraction (CNEAF) displayed the strongest cytotoxicity (IC_50_ = 48.81 ± 1.44 *µ*g/mL) to HCT-116 cells. CNEAF induced cell apoptosis, dissipated mitochondrial membrane potential, and elevated the reactive oxygen species (ROS) level, accompanied by the increase in expression of Bax and the decrease in expression of Bcl-2 and Bcl-X2, resulting in the activation of caspase−3, −9, −8, and −10. Upregulation in death receptor 5 was also detected. These data suggest that both intrinsic and extrinsic apoptosis pathways are involved in CNEAF-elicited effects. It is known that dysregulation of autophagy contributes to cancer; therefore, targeting autophagy would be a promising way for cancer treatment [25]. CNEAF also induced autophagy by the increase in LC-3 and decrease in p62 expression. CNEAF-induced elevation in the reactive oxygen species (ROS) level can be alleviated by N-acetylcysteine. In addition, N-acetylcysteine treatment also decreased both CNEAF-induced apoptosis and autophagy. These evidences showed that both apoptosis and autophagy elicited by CNEAF are ROS-dependent in HCT-116 cells [26].

### 2.4. Gastric Cancer

A novel polysaccharide-peptide complex CNP-1-2 with a molecular weight of 9.17 × 10^4^ Da was isolated by a series of complex purification methods from *C. nutans* leaves. CNP-1-2 displayed the strongest growth-inhibition effect on SGC-7901 human gastric cancer cells and was able to stimulate the activation of macrophages among all prepared polysaccharide fractions. CNP-1-2 consists of about 87.25% carbohydrate and 9.37% protein. The structural moieties of CNP-1-2 were determined by several approaches including monosaccharide analysis, methylation analysis, FT-IR, ^1^H NMR spectroscopy analysis, and atomic force micrograph (AFM) analysis [27]. Novel polysaccharide-peptide complex such as CNP-1-2 would open a new avenue to combat cancers, including gastric cancer [27].

### 2.5. Liver Cancer

A previous study has identified several phytochemicals and fractions, including *β*-sitosterol-3-O-*β* glucopyranoside, *β*-sitosterol-3-O-*β* glucoside, and subfraction F-III from the ethyl acetate extract of *C. nutans*. Among these isolates, the subfraction F-III exhibited strong cytotoxicity against HepG2 human hepatoma cells, with IC_50_ of 36.80 *µ*g/mL, but with no effect of this subfraction on antioxidant assay [28]. However, no further action mechanism was provided in this study. Thereafter, 30% ethanol extract (CN30) from aerial parts of *C. nutans* was produced and the active components in CN30 were furtherly fractionated and purified by using a bioassay that analyzes upregulation of the immune response of hepatoma cells–injected mice. High-performance liquid chromatography (HPLC) and mass spectrometry (LC/MS/MS) analyses showed that the main components of CN30 were identified to be (1) apigenin 6-C-*β*-D-glucopyranosyl-8-C-*α*-L-arabinopyranoside (known as shaftoside), (2) apigenin 6,8-C-*α*-L-pyranarabinoside, (3) orientin, (4) isoorientin, (5) vitexin, (6) isovitexin, and (7) gallic acid. CN30 at doses of 3 and 10 mg/kg induced 8.2% and 58.6% of inhibition in tumor size and tumor weight of the HepA xenograft model mouse. CN30 at dose of 10 mg/kg displayed stronger inhibition (58.6%) than that (37.1%) of the anticancer drug fluorouracil (20 mg/kg) in tumor weight. Further analyses showed that CN30 treatment induced significant apoptosis, decreased protein levels of proliferation markers PCNA and *p*-AKT, increased protein levels of apoptosis markers BAX and cleaved caspase-3, increased the number of IFN-*γ*^+^ T cells, decreased the number of IL-4^+^ T cells, and increased the levels of IFN-*γ* and interleukin-2 in serum of hepatoma-bearing mice. These data indicated that 30% ethanol extract (CN30) of *C. nutans* displays antitumor activity by augmenting the immune response and inducing apoptosis *in vivo* [29].

It has been reported that phytochemicals, including the total phenolic content (TPC) and total flavonoid content (TFC), were extracted from *C. nutans* by using organic solvents that include hexane, methanol, chloroform, and ethyl acetate and the cytotoxicity against HepG2 cells was tested. As compared to extracts of other solvents, methanol extract displayed strongest cytotoxic activity against HepG2 cells, with 74.17 ± 0.50% of inhibition at 100 *μ*g/mL after 24 hours of treatment. Further analyses showed that chloroform extract harbors highest total phenolic content (TPC), which is 119.29 ± 0.07 mg of gallic acid equivalent (GAE). Methanol extract contains highest total flavonoid content (TFC), which is 937.67 mg of butylated hydroxytoluene (BHT). These compounds could function as chemosensitizers [30] and provide sources of chemotherapeutic ingredients for cancer therapy in the future [31]. Quah also reported the antiproliferative activity of methanolic extract from *C. nutans* leaves on HepG2 cells with IC_50_ of 13.33 *μ*g/mL. However, the methanolic extract was much less toxic to normal 3T3 cells. The antiproliferative effect was possibly through inhibition in the enzymatic activities of two detoxifying enzymes CYP3A4 and CYP2E1 [19]. Another study reported the preparation of *C. nutans* extracts with five different solvents (hexane, chloroform, ethyl acetate, methanol, and water) and examined the anticancer activities on HepG2 cells. They found out that hexane and chloroform extracts inhibited cell viability of HepG2 cells, with the IC_50_ of 150 and 25 *μ*g/mL, respectively. Hexane extract induced formation of reactive oxygen species (ROS) and cell apoptosis. In addition, higher concentrations (≥100 *μ*g/mL) of hexane extract induced caspases 8, 9, and 3/7 activities. GC-MS analysis showed that 31 compounds exist in hexane extract, waiting for further analyses of the activity of each component on cancers. These studies suggested the involvement of both intrinsic and extrinsic apoptosis pathways underling the effects of hexane extract of *C. nutans* [32].

### 2.6. Lung Cancer

Fazil et al. evaluated the maximum yield and time of exhaustive extraction of flavonoids from *C. nutans* using Peleg's model and thereafter examined the antiproliferative activity of water extract on two-dimensional culture of A549 lung cancer cells. They obtained the predicted maximum extract density to be 29.20 ± 4.54 hours. However, the exhaustive time of extraction to have maximum flavonoids content was determined to be 18 hours and the best antiproliferative effects (IC_50_) on A549 cells was observed at 138.82 ± 0.60 *μ*g/mL. Such kinetics extraction modeling with modification could allow us to obtain the best timing with best yield for flavonoid water extraction from *C. nutans* or other herbal plants [33]. Another laboratory isolated hexane, chloroform, ethyl acetate, methanol, and water extracts from *C. nutans* and showed that hexane extract had highest cytotoxic activity to A549 cells, with the IC_50_ of 74 *μ*g/mL. Further evidences showed that hexane extract induced cell apoptosis, increased the percentage of cells at sub-G1 phase, and increased the levels of reactive oxygen species. Hexane extract also upregulated caspases 8, 9, and 3/7 activities at concentrations of more than 100 *μ*g/mL [32].

### 2.7. Lymphoma and Leukemia

Studies have shown that *C. nutans* chloroform extract displayed higher antiproliferative activities on K-562 human erythroleukemia cells (91.28 ± 0.03%) and Raji human Burkitt's lymphoma cells (88.97 ± 1.07%) at 100 *μ*g/mL, as compared to the rest of cell lines [10]. Moreover, Arullappan et al. found out that *C. nutans* petroleum ether extract displayed the strongest cytotoxic activity against K-562 cells with IC_50_ of 20.0 *μ*g/mL at 72h, as compared to other extracts [20]. However, the underlying mechanisms are not yet investigated. In addition to these cells, the activity of *C. nutans* extract to SUP-T1 human lymphoma cells and MOLT-4 leukemia cells was also reported by another laboratory. They obtained several fractions from *C. nutans* by extraction of leaf powder with different organic solvents and examined the effects of these extracts on various biological activities in SUP-T1 and MOLT-4 cells. The results showed that methanol-hexane-acetone extract (MHA) had the highest antiproliferative activities to SUP-T1 and MOLT-4 cells. Further studies showed that MHA increased cell apoptosis, reactive oxygen species, and calcium flux; arrested cell cycle at G2/M phase; and decreased the mitochondrial membrane potential and ER stress as evidenced by the increase in expression of CHOP and IRE-1*α* proteins, suggesting that MHA of *C. nutans* could possess anticancer activity in lymphoma and leukemia [34].

### 2.8. Head and Neck Cancer

Ng isolated C*. nutans* extracts of whole plants by hexane, chloroform, ethyl acetate, methanol, and water. They showed that hexane extract displayed strongest cytotoxicity to CNE-1 human nasopharyngeal carcinoma cells with IC_50_ of 116.7 *μ*g/mL. Hexane extract elicited cell apoptosis, increased the levels of reactive oxygen species and the percentage of cells at sub-G1 stage, and activated caspases 8, 9, and 3/7 at concentrations of ≥100 *μ*g/mL. Besides, ethyl acetate extract inhibited cell proliferation of CNE-1 cells at the concentration of 300 *μ*g/mL [32].

The extracts of *Clinacanthus nutans* are rich in flavonoids and polyphenols, which suffer from low solubility, poor permeability, and low bioavailability [35]. Nanoencapsulation is a good way of packaging drugs to overcome these drawbacks and increase drug efficacy [36, 37]. To this purpose, Yakop and colleagues reported the preparation of silver nanoparticle *Clinacanthus nutans* (AgNps-CN) of leaves aqueous extract and tested the anticancer activity including cytotoxicity, apoptosis, cell cycle progression, and expression of key proteins involving apoptosis in HSC-4 human oral squamous cell carcinoma cells. AgNps-CN inhibited the cell viability of HSC-4 cells with IC_50_ of 1.61 *μ*g/mL, while AgNps-CN is not harmful to normal 3T3-L1 cells at all tested concentrations. AgNps-CN induced cell apoptosis and cell cycle arrest at G0/G1 stage. Western blotting analyses showed that the ratio of Bax/Bcl-2 was increased by AgNps-CN. Due to its toxicity to HSC-4 cells and nontoxicity to normal cells, silver nanoparticles of *C. nutans* extracts could have the potential to treat cancers [12].

### 2.9. Skin Cancer

In order to know whether the extracts of plants from different geographical environment were different in their anticancer activities against skin cancer, Fong and his colleagues reported the production of crude methanol leaf extracts of *C. nutans* from different locations in Malaysia, Thailand, and Vietnam and examined the effects of extracts on cytotoxicity, apoptosis, and cell morphology of D24 melanoma cells. They found out that the extract from higher elevations with lower temperature was more toxic to D24 cells. Highest cytotoxic activities were found in extract from Chiang Dao and Chiang Mai, Thailand, with the half maximal effective concentration (E50) of 0.95 mg/mL for 24 hours and E50 of 0.77 mg/mL for 72 hours. Chiang Dao extract also induced apoptosis in biochemical and morphological analyses of D24 cells [38]. These data suggest that *C. nutans* extracts and extracts of plants in higher elevations and lower temperature could be more promising for the treatment of skin cancer.

## 3. Anticancer Activity of *C. nutans* (CN) Extract Combined with Current Anticancer Drug

### 3.1. Combined Effects of CN Extract with Cisplatin

Combination therapy with current anticancer drugs would be another way for *C. nutans* extracts to treat cancers. To this purpose, methanolic (MCN) and aqueous (WCN) extracts of *C. nutans* were obtained and combined treatment of extracts with anticancer drug cisplatin on MCF-7 breast cancer cells was tested. They found out that MCN displayed stronger cytotoxicity to MCF-7 cells than WCN. Most combined treatment of MCN and WCN extracts with cisplatin displayed strong antagonism. However, combined treatment of cisplatin with high concentration of MCN is additive (combination index = 1) against cancer cells [39].

### 3.2. Combined Effects of CN Extract with Gemcitabine

In addition to the cotreatment of *C. nutans* extracts with cisplatin described above, cotreatment with gemcitabine was also reported. Hii and his colleagues reported the isolation of four extracts, which include polar leaf extracts (LP), nonpolar leaf extracts (LN), polar stem extract (SP), and nonpolar stem extracts (SN) from *C. nutans*, and analyzed the antiproliferative activities of drug combination of *C. nutans* extracts with gemcitabine, on different cancer cell lines, including human breast, colorectal, lung, endometrial, nasopharyngeal, and pancreatic cancer cells by MTT assay. Among all cancer cell lines, AsPC1, BxPC3, and SW1990 pancreatic ductal adenocarcinoma (PDAC) cell lines were most sensitive to nonpolar stem extracts (SN). Addition of SN extract can reduce the dosage of gemcitabine with the same effect and can potentiate the killing activity of gemcitabine to PDAC by apoptosis through upregulation of Bax, as well as downregulation of bcl-2, cIAP-2, and XIAP levels in SW1990 and BxPC3 cells independent of the TRAIL-4 expression [40].

## 4. Concluding Remarks

*C. nutans* extracts display strong anticancer activities, including inhibition of cell proliferation, retardation of cell cycle progression, and induction of apoptosis and autophagy, in addition to its antiviral, antibacterial, and antioxidant effects. This review provides up-to-date information about anticancer activities and action mechanism of various *C. nutans* extracts, which were obtained by different solvents and different isolation methods, from different parts of the plants, different plant age, and plants of different geographical environment. Moreover, nanoencapsulation of *C. nutans* extract and combined treatment of *C. nutans* extracts with current anticancer drugs are evaluated in order to increase the drug efficacy and decrease possible side effects. These studies would shed light on the discovery of bioactive components of C*. nutans* and development of nature product-based drug using novel technologies [41], which could be applied for the therapy of various cancers in the future.

## Figures and Tables

**Table 1:** Anticancer activities of different extracts of *C. nutans* (CN for abbreviation).

Cancer type	Cancer cells	Extracts (plant parts, solvents, further fractionation)	Biological activities	Reference
(1) Breast cancer	MDA-MB-231 and MCF-7	(i) Six fractions isolated by chromatography of methanol crude extract of CN bark	(i) Decrease in cell viability of MDA-MB-231 and MCF-7 cells by fractions A12 and A17	Mutazah et al., 2019
		(ii) Two sulfur-containing compounds, including entadamide C and clinamide D were isolated from both fractions (A12 and A17)	(ii) IC_50_ of A12 to MDA-MB231 : 8.394 ± 0.086 *μ*g/mL	
			(iii) IC50 of A12 to MCF-7: 8.007 ± 0.043 *μ*g/mL	
			(iv) IC 50 of A17 to MDA-MB231 : 5.683 ± 0.064 *μ*g/mL	
			(v) IC50 of A17 to MCF-7: 5.048 ± 0.083 *μ*g/mL	
			(vi) Caspase-3 is the cellular target of both entadamide C and clinamide D by molecular docking analyses	
	4T1 breast tumor-bearing mice	(i) Methanol extract of CN leaves	(i) Effective reduction in the number of mitotic cells, tumor weight, and tumor volume of 4T1 breast cancer model mice by high-dosage (1000 mg/kg) extract	Nik Abd Rahman et al., 2019
	MCF-7	(i) Methanol and ethyl acetate extracts of CN roots	(i) Inhibition in proliferation of MCF-7 cells by methanol extract (IC_50_ : 35 *μ*g/mL) and ethyl acetate extract (IC_50_ : 30 *μ*g/mL)	Teoh et al., 2017
		(ii) 14 compounds and 7 compounds were isolated from ethyl acetate root extract and methanol root extract respectively	(ii) Noncytotoxic to NIH 3T3 cells at all concentration by methanol and ethyl acetate extracts	
			(iii) Methanol and ethyl acetate extracts: induction of apoptosis and decrease in *BCL2* expression	
			(iv) No change in *BAX* expression	
			(v) Ethyl acetate extract: decrease in the mitochondrial membrane potential	
	MCF-7	(i) Methanol crude extract and further fractionation by n-hexane, dichloromethane, chloroform, n-butanol, and water of CN leaves	(i) Crude extract (CN-Crd) itself had the strongest antioxidant scavenging activity, while water (CN-Aqu) fraction possessed the highest amount of total phenolic content (TPC) and total flavonoid content (TFC)	Ismail et al., 2017
		(ii) Identification by GC-MS of dichloromethane fraction (CN-Dcm) yields 14 compounds. Two most abundant compounds are linolenyl alcohol (29.10% at 12.023 min) and palmitic acid (23.84% at 11.133 min)	(ii) Hexane fraction (CN-Hex) displayed the highest antiproliferative effects on MCF-7 cells, with the IC_50_ of 50.34 ± 0.11 *μ*g/mL, followed by the dichloromethane fraction (CN-Dcm) (IC_50_ : 65.95 ± 0.14 *μ*g/mL)	
	MDA-MB-231	(i) Methanolic extract from *C. nutans* leaves	(i) Antiproliferative activity of methanolic extract to MDA-MB-231 cells (IC_50_ : 18.67 *μ*g/mL)	Quah et al., 2017
			(ii) The antiproliferative effects were possibly through the inhibition of CYP3A4 and CYP2E1 activities	

(2) Cervical cancer	HeLa	(i) Methanol-hexane, methanol-dichloromethane (DCM), and methanol-water extracts of *C. nutans*	(i) Antiproliferative activities to HeLa cells for all extracts	Haron et al., 2019
		(ii) At least 28 compounds in DCM by GC-MS analysis and most of them were fatty acids	(ii) DCM: Highest antiproliferative activity (IC_50_ = 70 *μ*g/mL) at 48 hours	
			(iii) DCM: induction of apoptosis and cell cycle arrest at S phase	
	HeLa	(i) Aqueous and methanol extracts of CN leaves	(i) Aqueous extract: strong cytotoxic effects (IC_50_ = 13 ± 0.82 *μ*g/mL)	Yusmazura et al., 2017
			(ii) Aqueous extract: induction of apoptosis	
			(iii) No cytotoxicity to normal kidney cell lines (vero) by both extracts	
			(iv) No significant cytotoxic effect of methanol extract with no IC_50_ detected	
	HeLa	(i) The methanol extracts of leaves and buds of different plant ages, including 1-month-, 6-month-, and 1-year-old plant materials of *C. nutans*	(i) Highest 1,1-diphenyl-2-picrylhydrazyl (DPPH) activity in the extract of 1-year-old buds, with 50% of free radical scavenging (IC_50_) values of 64.6 *μ*g/mL	Ghasemzadeh et al., 2014
		(ii) Highest total flavonoid (TF) (6.32 mg/g dry weight [DW]) and total phenolic (TP) compounds (18.21 mg/g DW) in 6-month-old buds	(ii) Higher ferric reducing antioxidant power (FRAP) activity in 6-month-old buds (488 *μ*M of Fe(II)/g) than in 1-year-old buds (453 *μ*M of Fe(II)/g)	
		(iii) Highest contents of caffeic acid (0.307 mg/g DW) and gallic acid (5.96 mg/g DW), phenolic acids in extracts of 1-year- and 6-month-old buds respectively	(iii) Decrease in cell viability of HeLa cells (IC_50_ = 56.8 *μ*g/mL) by extracts of 6-month-old buds	
		(iv) Highest chalcone synthase (CHS, EC 2.3.1.74) activity in 6-month-old buds (9.5 nkat/mg protein)		
	HeLa	(i) Petroleum ether, ethyl acetate, and methanol crude extracts of *Clinacanthus nutans* by bioassay-guided fractionation	(i) Strongest cytotoxic activity against HeLa cells (IC_50_ = 18 *μ*g/mL) by petroleum ether extract	Arullappan et al., 2014
			(ii) Highest radical scavenging activity in petroleum ether extract	

(3) Colorectal cancer	HCT-116	(i) Ethanol extract, hexane, ethyl acetate, and aqueous fractions of *C. nutans* leaves	(i) Strongest cytotoxicity (IC_50_ = 48.81 ± 1.44 *μ*g/mL) to HCT-116 cells by *C. nutans* ethyl acetate fraction (CNEAF)	Wang et al., 2017
			(ii) CNEAF: induction of apoptosis; decrease in mitochondrial membrane potential; increase in reactive oxygen species (ROS); increase in bax expression; decrease in Bcl-2 and Bcl-X2 expression; activation of caspase−3, −9, −8, and −10	
			(iii) Upregulation in death receptor 5 expression by CNEAF	
			(iv) Autophagy induction by CNEAF (increase in LC-3 level; decrease in p62 level)	
	HCT-116	(i) Ethanolic, methanolic, 50% ethanolic, 50% methanolic, and water extracts of *C. nutans* leaves	(i) All crude extracts at concentrations of 200 and 100 *μ*g/mL: no significant cytotoxicity on tested cell lines	Esmailli et al., 2013
			(ii) Fractions 3, 4, 14, and 16 of methanolic extract displayed significant cytotoxicity to HCT-116 cells at the concentration of 200 *μ*g/mL	
			(iii) Fraction 14 : 84 ± 1.1% of growth inhibition at 100 *μ*g/mL; other fractions <50% of inhibition	

(4) Gastric cancer	SGC-7901	(i) Isolation of a novel polysaccharide-peptide complex CNP-1-2 with molecular weight of 9.17 × 10^4^ Da from CN leaves by a combination of methods	(i) Inhibition in cell viability of SGC-7901 cells concentration dependently (50, 100, 200 *μ*g/mL) by CNP-1-2	Huang et al., 2016
		(ii) CNP-1-2 consists of about 87.25% of carbohydrate and 9.37% of protein		
		(iii) Structure of CNP-1-2 was solved by methylation analysis, FT-IR, and ^1^H NMR spectroscopy analysis		

(5) Liver cancer	HepG2	(i) Five solvent extracts (hexane, chloroform, ethyl acetate, methanol, and water) of CN whole plants	(i) Both hexane and chloroform extracts displayed cytotoxic activity to HepG2 cells (IC_50_ of hexane extract: 150 *μ*g/mL; IC_50_ of chloroform extract: 25 *μ*g/mL)	Ng et al., 2017
			(ii) Hexane extract: induction of apoptosis and increase in the percentage of cells at sub-G1 stage; increase in the levels of ROS	
			(iii) Hexane extract: activation of caspases 8, 9, and 3/7 at high concentrations (≥100 *μ*g/mL)	
	HepG2	(i) Methanolic extract from *C. nutans* leaves	(i) Antiproliferative activity of methanolic extract to HepG2 cells (IC_50_: 13.33 *μ*g/mL)	Quah et al., 2017
			(ii) The antiproliferative effects were possibly through the inhibition of CYP3A4 and CYP2E1 activities	
	HepG2	(i) The extraction of phytochemicals, including the total phenolic content (TPC) and total flavonoid content (TFC), from *Clinacanthus nutans* by organic solvents (hexane, methanol, chloroform, and ethyl acetate)	(i) Cytotoxic activity of extracts to HepG2: methanol (IC_50_: 43.9367 *μ*g/ml)> chloroform (IC_50_: 55.6112 *μ*g/ml)> ethyl acetate (IC_50_: 62.0655 *μ*g/ml)> hexane extract (IC_50_: 68.3807 *μ*g/ml)	Hamid and Yahaya, 2016
		(ii) Chloroform extract: with highest total phenolic content (119.29 mg of gallic acid equivalent (GAE))		
		(iii) Methanol extract: with highest total flavonoid content (937.67 mg of butylated hydroxytoluene (BHT))		
	HepA xenograft mice model	(i) 30% ethanol extract (CN30) of CN	(i) CN30: decrease in tumor weight and volume	Huang et al., 2015
		(ii) 7 compounds were identified from CN30, including gallic acid, shaftoside, isoorientin, orientin, isovitexin, vitexin, apigenin 6, 8-di-*C*-*α*-L-arabinopyranoside	(ii) Apoptosis induction of hepatoma cells *in vivo*	
			(iii) Decrease in the expression of proliferation markers PCNA and *p*-AKT	
			(iv) Increase in expression of cleaved caspase-3; decrease in expression of BAX and Bcl2	
			(v) Increase in the number of IFN-*γ*^+^ T cells and decrease in the number of IL-4^+^ T cells	
			(vi) Increase in the serum IFN-*γ* and interleukin-2 levels	
	HepG2	(i) *β*-sitosterol-3-O- *β* glucopyranoside, *β*-sitosterol-3-O- *β* glucoside, and subfraction F-III were extracted and identified from ethyl acetate extract	(i) Strong cytotoxicity to HepG2 (IC_50_: 36.80 *µ*g/mL) by subfraction F-III	Dan, 2014
			(ii) no effect on anti-oxidant assay by sub-fraction F-III	

(6) Lung cancer	A549	(i) Extracts by five solvents (hexane, chloroform, ethyl acetate, methanol, and water) of CN whole plants	(i) Highest cytotoxic activity of hexane extract to A549 cells (IC_50_: 74 *μ*g/mL)	Ng et al., 2017
			(ii) Hexane extract: induction of apoptosis and increase in the percentage of cells at sub-G1; increase in the levels of ROS	
			(iii) Hexane extract: upregulation of caspases 8, 9, and 3/7 activities at high concentrations (≥100 *µ*g/mL)	
	A549	(i) Water extract of CN using kinetic extraction modeling to obtain maximum yield of flavonoids	(i) The best antiproliferative effects (IC_50_): 138.82 ± 0.60 *µ*g/mL on two-dimensional cell culture of A549 cells	Fazil et al., 2016
		(ii) 18 hours of extraction was determined to obtain the maximum content of flavonoids		

(7) Lymphoma and leukemia	MOLT-4 and SUP-T1	(i) Methanol extract of leaves of CN, then extracted by hexane (MH), methanol (M), ethyl acetate (ME), and butanol (MB) respectively	(i) Strong decrease in the cell viability of MOLT-4 cells by the acetone extract (MHA)	Lu et al., 2018
		(ii) MH fraction was further extracted by hexane (MHH) and acetone (MHA)	(ii) Decrease in the cell viability of SUP-T1 cells dose dependently	
			(iii) MHA: induction of apoptosis; decrease in the mitochondrial membrane potential; increase in the levels of ROS; increase in calcium ions in SUP-T1 cells	
			(iv) MHA: increase in protein levels of active caspase−3, −7, and −8 in SUP-T1 cells	
			(v) MHA: decrease in Bcl-xl and Bcl-2 expression; increase in Bim, Bak, and cytochrome C expression in SUP-T1 cells	
			(vi) MHA: induction of ER stress (increase in CHOP and IRE-1*α* expression) in SUP-T1 cells	
			(vii) MHA: decrease in hexokinase II expression in SUP-T1 cells	
			(viii) MHA: increase in TNF-1*α*, NF-*κ*B, and DR5 expression at concentration of 50 *μ*g/mL in SUP-T1 cells	
	K562	(i) Chloroform, methanol, and water extracts of CN leaves	(i) Highest antiproliferative effect (91.28 ± 0.03%) of chloroform extract at 100 *μ*g/ml to K-562 cells	Yong et al., 2013
	Raji	(i) Chloroform, methanol, and water extracts of CN leaves	(i) Highest antiproliferative effect (88.97 ± 1.07%) of chloroform extract at 100 *μ*g/ml to Raji cells	Yong et al., 2013

(8) Head and neck cancer	HSC-4	(i) Silver nanoparticles (AgNps-CN) of CN leaves extract	(i) Cytotoxicity of AgNps-CN to HSC-4 cells (IC_50_: 1.61 ± 0.14 *μ*g/mL)	Yakop et al., 2018
			(ii) Nontoxicity of AgNps-CN to 3T3-L1 cells at high concentration (3.00 *μ*g/mL)	
			(iii) AgNps-CN: induction of apoptosis and cell cycle arrest at G1 phase of HSC-4 cells	
			(iv) AgNps-CN: increase in Bax expression; decrease in Bcl-2 expression	
	CNE-1	(i) Five solvent extracts (hexane, chloroform, ethyl acetate, methanol, and water) of CN whole plants	(i) Highest cytotoxic activity of hexane extract to CNE-1 cells (IC_50_: 116.7 *μ*g/mL)	Ng et al., 2017
			(ii) Antiproliferative activity of ethyl acetate extract to CNE-1 cells at 300 *µ*g/mL	
			(iii) Hexane extract: induction of apoptosis and increase in the percentage of cells at sub-G1, increase in ROS	
			(iv) Hexane extract: activation of caspases 3/7, 8, and 9 at the concentrations of ≥100 *µ*g/mL	

(9) Skin cancer	D24	(i) The crude methanol extracts of CN from 11 different locations in Malaysia, Thailand, and Vietnam	(i) More toxicity of extracts from higher elevations with lower temperature to D24 melanoma cells	Fong et al., 2016
			(ii) Highest cytotoxic activities were found in Chiang Dao, Chiang Mai, Thailand; the half maximal effective concentration (EC_50_): 0.95 mg/mL, 24 hours; EC_50_: 0.77 mg/mL, 72 hours	
			(iii) Induction of apoptosis by Chiang Dao extract	

**Table 2:** Anticancer activity of *C. nutans* (CN) extract combined with current anticancer drug.

Cancer cells	Extracts (plant parts, solvent, fractionation)	Current anticancer drug	Biological activities	Reference
A total of 23 human cancer cell lines:	(i) Polar leaf extract (LP), nonpolar leaf extract (LN), polar stem extract (SP), and nonpolar stem extract (SN) of CN	Gemcitabine	(i) AsPC1, BxPC3 and SW1990 pancreatic ductal adenocarcinoma (PDAC) cell lines were found to be the most sensitive to SN extract	Hii et al., 2019
(i) Human breast cancer cells (MCF-7, MDA-MB-231, MDA-MB-468, HCC38)			(ii) Addition of SN extract can reduce the dose of gemcitabine in PDAC
			(iii) Drug combination can potentiate the killing activity of gemcitabine to PDAC by apoptosis (increase in Bax expression; decrease in Bcl-2, cIAP2, and XIAP expression) in SW1990 and BxPC3 cells
(ii) Colon cancer cells (HCT-116, HT29, SW48, Caco2)			(iv) The killing activity is independent of the TRAIL-4 expression
(iii) Lung cancer cells (A549, NCI-H1299, NCI-H23, Calu-1)
(iv) Endometrial cancer cells (AN3-CA, HEC-1-A, HEC-1-B, RL95–2)
(v) Nasopharyngeal, cancer cells (CNE-1, HK1, SUNE1, TWO1)
(vi) Human pancreatic ductal adenoma, PDAC (AsPC1, BxPC3, SW1990, Panc 10.05).

Human breast cancer cells (MCF-7)	Methanol extract (MCN) and aqueous extract (WCN) of CN	Cisplatin	(i) Cytotoxicity to MCF-7 : MCN > WCN	Abd Mutalib et al., 2019
			(ii) Both extracts showed strong antagonism when used in combination with cisplatin
			(iii) Only combined treatment of cisplatin with high concentration of MCN is additive (combination index = 1) against cancer cells

## Abbreviations

*C. nutans* (CN): *Clinacanthus nutans*.
